# Botulinum toxin injection shows promise in nail psoriasis: A comparative randomized controlled trial

**DOI:** 10.1016/j.jdin.2024.03.021

**Published:** 2024-04-30

**Authors:** Premjit Juntongjin, Suthima Srisinlapakig, Sunatra Nitayavardhana

**Affiliations:** Division of Dermatology, Chulabhorn International College of Medicine, Thammasat University, Pathumthani, Thailand

**Keywords:** botulinum toxin, injection, nail psoriasis, randomized controlled trial, RCT, treatment

## Abstract

**Background:**

Nail psoriasis remains a challenging condition with limited satisfaction from current treatments. An increasing number of neuropeptides were reported in psoriatic tissue.

**Objective:**

To compare the efficacy of botulinum toxin A (BoNT-A) injection, triamcinolone acetonide (TA) injection, topical combination of vitamin D/steroid (VitD/steroid) and placebo in improving nail psoriasis.

**Methods:**

A 24-week randomized intraindividual comparative-controlled study involved participants with at least 4 psoriatic fingernails, each with a total target nail psoriasis severity index (NAPSI) score of at least 3 points. Nails were randomly received different treatments; intralesional BoNT-A injection at baseline, intralesional TA at baseline and eighth week, daily topical VitD/steroid application for 16 weeks and placebo.

**Results:**

Evaluation of 64 psoriatic fingernails showed a 40% reduction in the total target NAPSI score at 24 weeks following BoNT-A injection (*P* = .001). BoNT-A significantly improved nail bed lesions more than TA and topical VitD/steroid (*P* = .038), with no reported serious adverse effects.

**Limitations:**

Relatively small sample size; hand hygiene during the COVID-19 pandemic may interfere NAPSI score evaluation.

**Conclusions:**

BoNT-A injection emerges as a promising and effective therapy for nail psoriasis, providing sustained efficacy lasting up to 6 months with a single injection.

**Capsule Summary**

• Botulinum toxin A holds promise as a potential treatment for nail psoriasis through its neuromodulatory effects.
• A single injection of botulinum toxin A demonstrates long-lasting efficacy in nail psoriasis, particularly for lesions on the nail bed.

## Introduction

Psoriasis impacts approximately 3% of the adult population.^1^ Nail psoriasis has been noticed up to 80% of cutaneous psoriasis patients at some point in their lives.^2^ Nail changes can even occur in 5% to 10% of individuals with minimal or no visible skin psoriasis. Additionally, nail psoriasis is considered a remarkable physically and emotionally distressing affecting patients’ quality of life.^3^

The therapeutic strategies for the management of nail psoriasis are limited. Regarding to the recommendations for the treatment of nail psoriasis in adults with no or minimal skin involvement,^4^ the treatment depends on the number and location of nail involvement. Intralesional steroids and topical combinations of vitamin D and steroid (VitD/steroid) often play a role in the treatment. However, the efficacy of topical medications is often compromised by limited drug absorption, resulting in unfavorable and refractory outcomes. Intralesional steroids injection necessitate multiple treatment sessions and are associated with pain. Systemic therapies, including conventional immunosuppressants, oral retinoids, biologics, and small molecules are typically considered for cases where >3 nails are affected or when the condition significantly impairs the patients’ quality of life; nevertheless, adverse reactions are frequently concerned.

The exact pathogenesis of nail psoriasis remains elusive. Over the past decade, evidences from immunologic and genetic researches have suggested predominant involvement of IL-17 and IL-23 in causal immunologic pathways.^5^ Nonetheless, neurogenic inflammation, one of the pathogeneses of psoriasis, is not frequently described. Previous studies^6^, ^7^, ^8^ indicated an elevated concentration of nerve fibers and increased level of neuropeptides, including calcitonin gene-related peptide and substance P in psoriatic skin. Neuropeptides’ expression potentially contributes to acanthosis and infiltration of immune cells into the psoriatic skin.

Botulinum toxin A (BoNT-A) treatment has shown promising results in both animal models and clinical efficacy of cutaneous psoriasis. Abobotulinum toxin A (Abo-BoNT-A), in particular, has exhibited efficacy in improving acanthosis and reducing neuromodulators in a mouse model of psoriasiform dermatitis.^9^ Several studies^10^, ^11^, ^12^, ^13^, ^14^ revealed the effectiveness of BoNT-A in recalcitrant plaque-type and inverse psoriasis. Recently, there was a report^15^ of 2 successful cases following a single Abo-BoNT-A injection in psoriasis nails.

Given the significant challenges in treating psoriatic nails, this study aims to investigate the potential of intralesional BoNT-A injection as a novel therapeutic approach for nail psoriasis.

## Materials and methods

### Research design

This study was a prospective, randomized, evaluator-blinded, intraindividual controlled trial. Before the enrollment, all participants were thoroughly informed about the methodology, potential risks and benefits, and any possible adverse events associated with the study.

### Participants

Nail psoriasis was clinically diagnosed. Each participant exhibited a minimum of 4 psoriatic fingernails, with a target nail psoriasis severity index (NAPSI) score of at least 3 points per nail. Individuals with allergies to botulinum toxin products, vitamin D, corticosteroid, cow’s milk protein, or lidocaine were excluded. The exclusion criteria also encompassed an individual with neuromuscular disorders or peripheral motor neuropathic diseases, pregnancy, and breastfeeding. Participants who had undergone previous topical nail therapies were required to discontinue the treatment prior for at least 2 weeks before enrollment. After informed consent, comprehensive data collection including patients’ personal history, photographs, dermatoscopic examinations of fingernails and physical examinations were conducted.

### Treatment protocol

**Fig 1:**
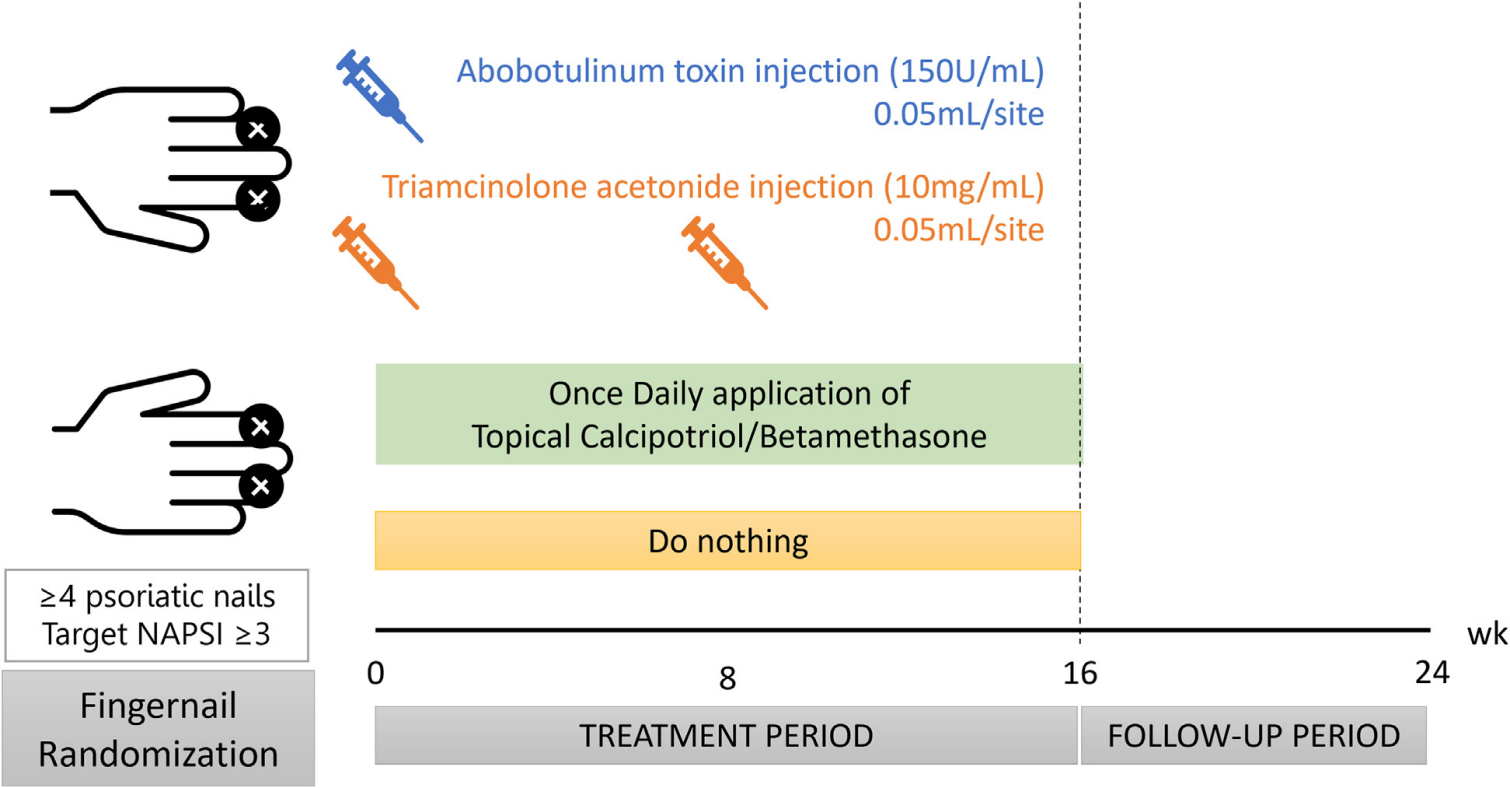
Study protocol.

Each patient underwent 4 distinct treatment modalities, categorized into BoNT-A, triamcinolone acetonide (TA), topical VitD/steroid and placebo. These groups were randomly matched to each psoriatic nail using a computer generator. In BoNT-A group, the psoriatic nail was injected intralesional with Abo-BoNT-A (Dysport) at 0.05 mL/site (7.5 U/site) at week 0. TA group received intralesional injections of TA (10 mg/mL) at 0.05 mL/site in a psoriatic nail at weeks 0 and 8. VitD/steroid participants applied topical calcipotriol/betamethasone dipropionate ointment (Daivobet) once daily to a psoriatic nail for 16 weeks. In placebo, nails did not receive any treatment for a duration of 24 weeks (Fig 1).

Intralesional injections were administered employing the modified De Berker’s technique.^16^ The number of the injection sites varied depending on where the psoriasis was located. Patients received intradermal injections at 2 sites on the proximal nailfold for nail matrix lesions and/or 2 sites on the nail bed for nail bed lesions. Digital nerve block with 2% lidocaine HCl and the application of topical 5% lidocaine/prilocaine anesthetic cream were performed for all patients. The injection was carried out using 30-gauge needles.

### Efficacy and safety assessment

A blinded evaluator assessed the NAPSI scores in 4 recruited fingers at baseline (week 0) and week 8, 16, and 24 using a dermatoscope. Each nail was visually divided by imaginary horizontal and longitudinal lines into quadrants. An individual quadrant was given a score for nail matrix psoriasis, including pitting, leukonychia, red spots in the lunula and nail plate crumbling (0-4), and nail bed psoriasis, including onycholysis, splinter hemorrhages, oil drop discoloration, and nail bed hyperkeratosis (0-4) depending on the presence of any features of nail psoriasis in that quadrant. The sum of these scores provided a total NAPSI score for each nail (0-16).

During each injection, participants rated their pain on a 0 to 10 scale (0 = no pain, 10 = worst pain) for both group A and B injections. At every follow-up visit, adverse reactions were evaluated.

### Statistical analysis

Data were analyzed using the IBM Statistical Package for the Social Sciences (SPSS, 27.00 version). Descriptive and inferential analyses were employed to interpret the gathered data. The efficacy of BoNT-A injection, TA injection, topical VitD/steroid and the control group was assessed through the Friedman test and Wilcoxon’s signed rank test. The NAPSI scores, categorized into total, matrix, and nail bed score were compared.

## Results

This study initially included 18 participants with psoriatic nail involvement, encompassing a total of 72 fingernails. After the first visit, 2 participants discontinued due to loss of follow-up and required systemic treatment from the worsening cutaneous psoriasis. Consequently, 16 patients with a total of 64 psoriatic nails successfully completed the 24-week follow-up.

The mean age of the patients was 45 years old (30-71). More than half of the participants were male. All participants had plaque-type psoriasis, with a mean psoriasis area and severity index (PASI) score of 4.7 and an average disease duration of 11 years. The mean body mass index was within the overweight range. The demographic characteristics of the participants were shown in Table I.

**Table I:** Demographic characteristics of the participants

	n
Number of participants	16
Number of nails included	64
Age, y (range)	45.6 (30-71)
**Sex** (%)	
Male	75
Female	25
Type of psoriasis (%)	
Plaque-type psoriasis	100
PASI score (mean ± SD)	4.7 ± 2.1
Total target NAPSI score (mean ± SD)	7.76 ± 2.8
Duration of disease, y (mean ± SD)	11.3 ± 6.5
BMI, kg/m^2^ (mean ± SD)	26.9 ± 4.5

*BMI*, Body mass index; *NAPSI*, nail psoriasis severity index; *PASI*, psoriasis area and severity index.

### Total target NAPSI change

**Fig 2:**
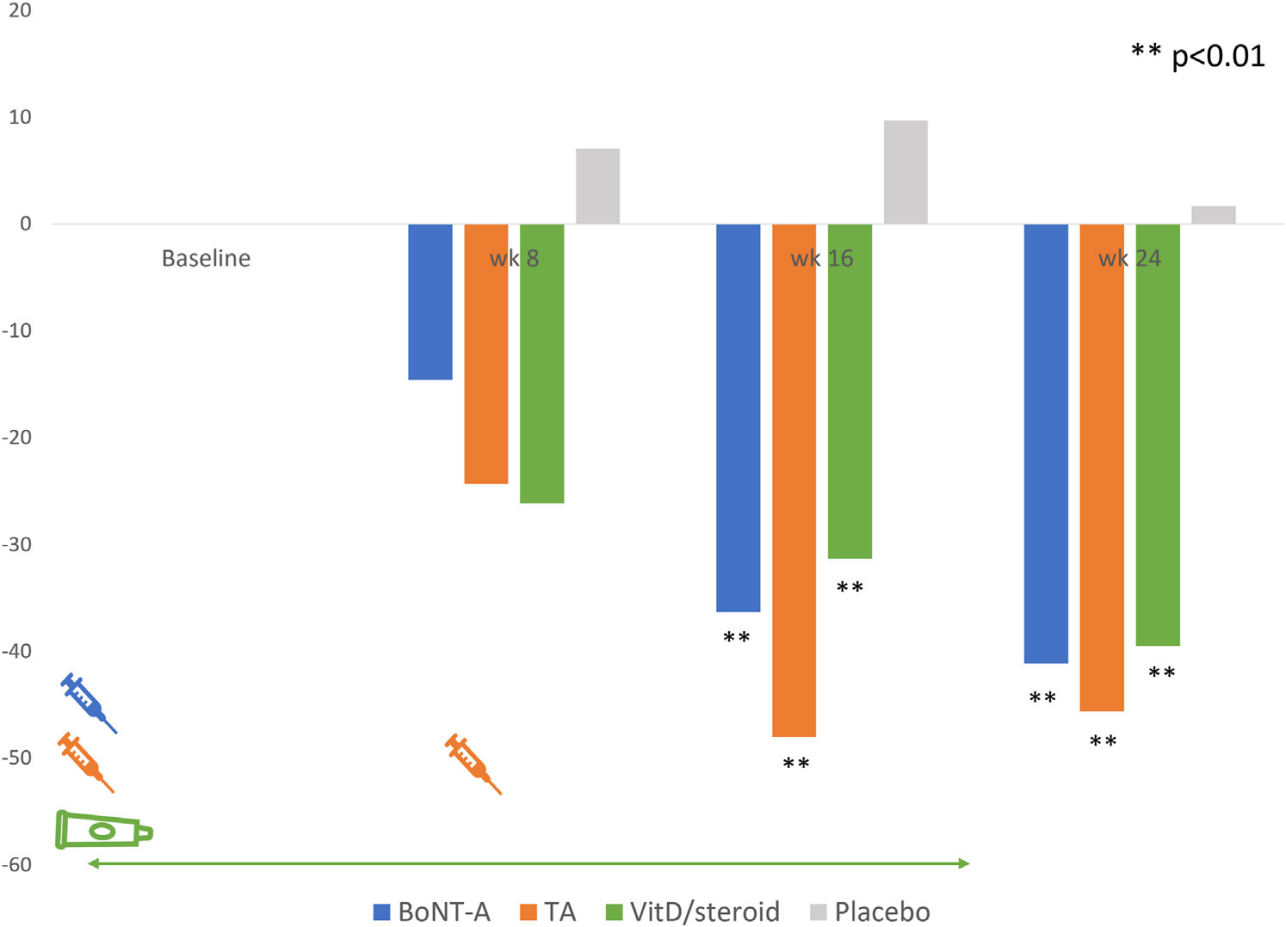
Total target NAPSI change (%). *NAPSI*, Nail psoriasis severity index.

At baseline, the mean target NAPSI was at approximately 7.7. There was no significant difference among the assigned regimens (Fig 2). By the 16th week, all 3 regimens exhibited significant nail improvement compared with the baseline (*P* < .01). Following a single BoNT-A injection, the total target NAPSI gradually decreased, reaching a nearly 40% reduction (*P* = .008). With every 8-week injection of TA, there was a 50% reduction in the total target NAPSI (*P* = .001), whereas the combination of topical VitD/steroid showed a roughly 30% change (*P* = .003). Notably, at the follow-up period of week 24, the BoNT-A group continued to display a positive trend in nail improvement, whereas the TA group appeared stable. However, there was no significant difference between the BoNT-A and TA at any time point.

### Nail bed target NAPSI change

**Fig 3:**
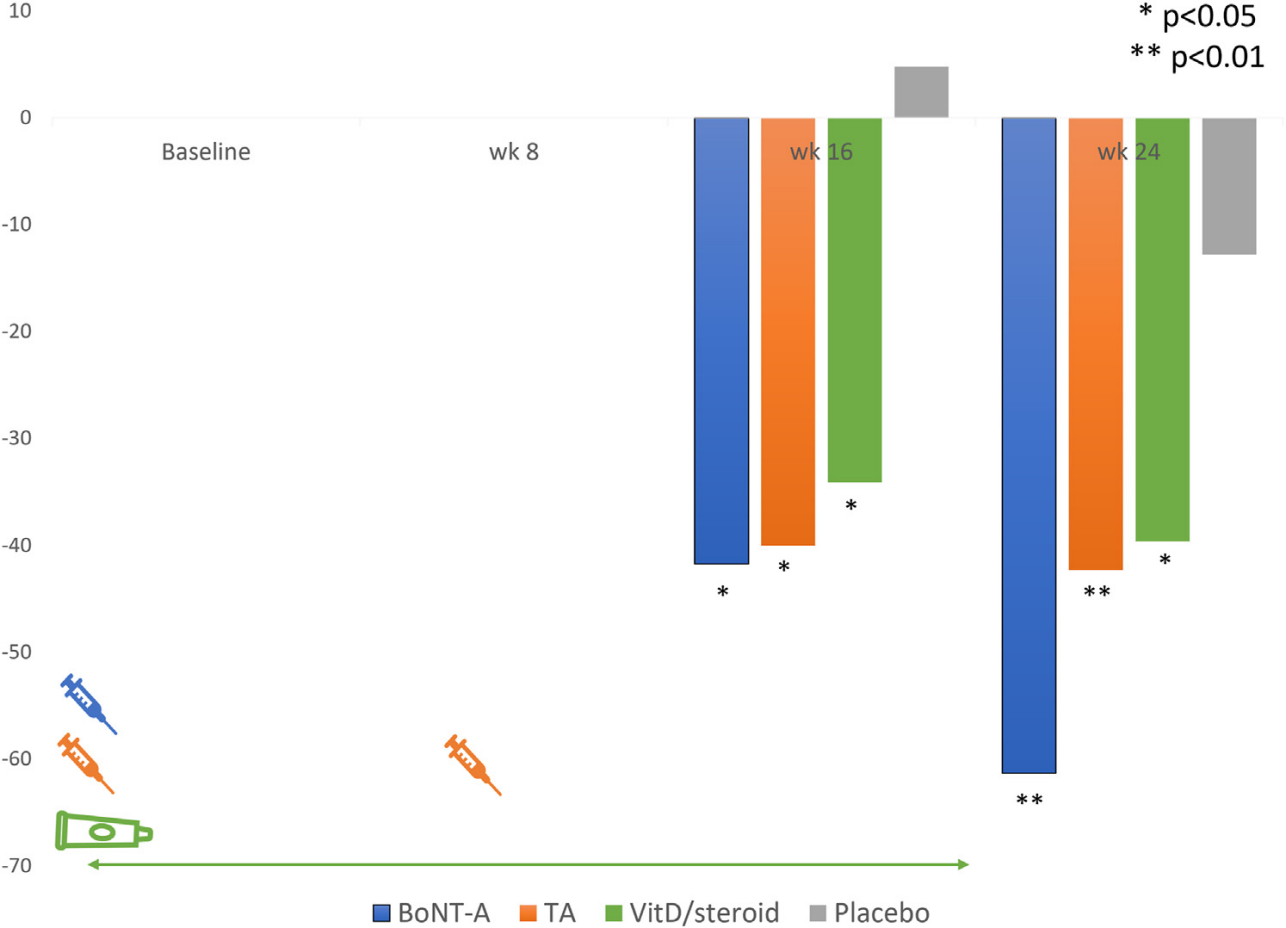
Nail bed target NAPSI change (%). *NAPSI*, Nail psoriasis severity index.

At both 16th and 24th weeks, all 3 treatment approaches resulted in a significant reduction of nail bed target NAPSI (*P* < .05) (Fig 3). By the 16th week, both BoNT-A (*P* = .02) and TA (*P* = .01) injections demonstrated an approximately 40% reduction in nail bed target NAPSI. Interestingly, there was a significant divergence in nail bed changes among all treatments at week 24 (*P* = .002). The improvement of nail bed could reach up to 60% following a single injection of BoNT-A (*P* = .003) density, whereas with the TA injection showed a stable outcome since week 16. The difference in change during the follow-up period was significant between the BoNT-A and TA regimens (*P* = .038).

### Adverse reactions

There was no statistically significant difference in pain scores between the BoNT-A and TA injections, with both regimens averaging a score of 5 out of 10. After the BoNT-A injection, 1 participant experienced pain lasting for 6 hours, which spontaneously resolved later. In another participant who received the TA injection under the nail plate, a hematoma was developed but gradually disappeared within 2 weeks. No serious adverse reactions to either the injections or topical medication were reported throughout the study.

## Discussion

**Fig 4:**
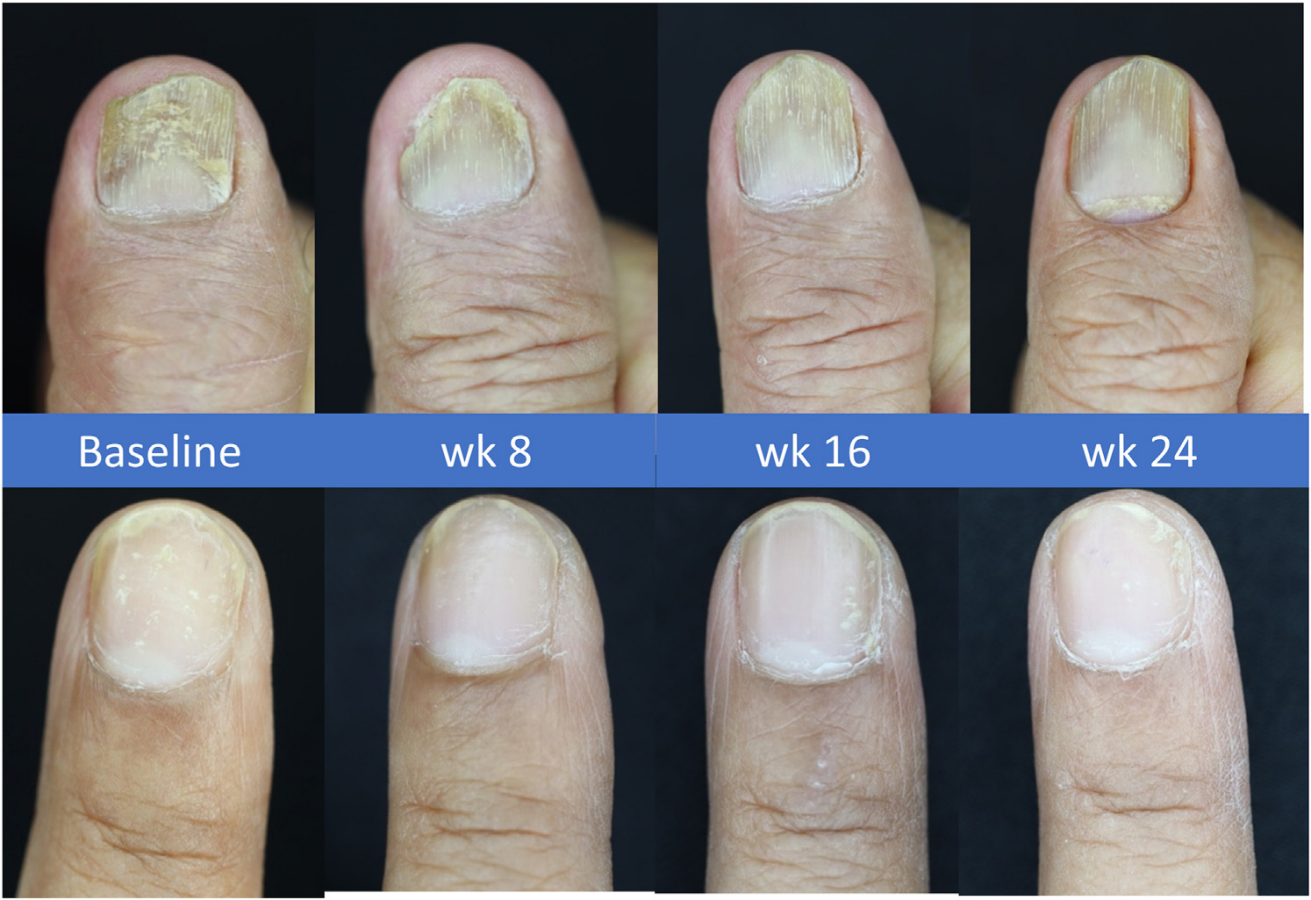
Clinical improvement following a single BoNT injection. *BoNT*, Botulinum toxin.

Nail psoriasis remains a challenging dermatologic condition, with available treatments offering limited satisfaction. In this study, all treatment regimens showed a gradual reduction in the total target NAPSI score by the 16th week. The BoNT-A group exhibited a continuous decrease in the score, whereas the TA group remained stable at the 24th week of the study. Therefore, a single injection of intralesional BoNT-A led to a 40% improvement in nail psoriasis in the 6-month period (Fig 4).

Focusing on nail bed changes, BoNT-A consistently demonstrated a reduction in target nail bed NAPSI from baseline to the 24th week. Nail bed improvement reached up to 60% following the single injection of BoNT-A at baseline, whereas the TA group exhibited a stable outcome from the fourth month onward. The difference between botulinum toxin and TA injections during the follow-up period was significant. These findings contribute to our understanding of product half-life, highlighting that BoNT-A can provide long-lasting efficacy for up to 6 months, whereas steroids last only 2 months.

Previous research had highlighted the efficacy of both Abo-BoNT-A and onabotulinum toxin A in treating cutaneous psoriasis. In our study, a singular injection of botulinum toxin revealed a significant improvement in nail psoriasis, amounting to approximately 40%. These results are concordant with other studies^12^^,^^14^ in recalcitrant cutaneous psoriasis.

Although, the exact mechanisms of BoNT-A on psoriasis remain unclear, a previous study suggested that the reduction in cutaneous psoriasis severity might be associated with a decrease in substance P and calcitonin gene-related peptide-immunoreactive nerve expression, coupled with an increase in epidermal nerve fiber density.^12^ In our study, significant benefits of intralesional BoNT-A were observed in nail bed lesions. This could be attributed to the well-vascularized nature of nail beds, which offers a higher concentration of nerve endings compared with the nail matrix. As a result, the nail beds may experience robust neuromodulatory effects from BoNT-A. Due to the limited space in both nail bed and nail matrix, Abo-BoNT-A, characterized by its small complex size and easy spread, was preferred for its potentially to affect a broader area.

In this study, the injection of TA every 8 weeks resulted in a reduction of approximately 48% in the total target NAPSI score, which comparable to previous studies,^16^^,^^17^ where the score decreased by 45% and 42.25%, with the injections every 2 months and every month, respectively. The improvement of nail psoriasis through the once-daily application of a topical VitD/steroid in our study demonstrated lower efficacy compared with previous studies.^18^, ^19^, ^20^ This difference in effectiveness may be since our study was conducted during the COVID-19 pandemic, a period when individuals increased handwashing and sanitizing, affecting the treatment’s ability to penetrate the skin.

To our knowledge, this study is the first randomized intraindividual controlled trial to demonstrate the efficacy of BoNT-A injection compared with intralesional steroid injection and the topical VitD/steroid that are the first line of treatment for nail matrix and nail bed lesions in nail psoriasis. This design ensures to eliminate potential biases caused by patient differences or external factors. The use of target NAPSI scores allows for a precise evaluation of the severity of each individual nail, and a follow-up period was implemented to assess disease relapse posttreatment discontinuation.

However, there are limitations to this study, including the relatively low number of participants and the short duration of the study period, suggesting that the effects of BoNT-A may potentially last longer than the observed 6 months. Additionally, the study was conducted during the COVID-19 era, introducing the possibility that COVID-19 vaccination or infection and increased hand hygiene practices. These factors may have affected psoriasis severity and potentially interfered with NAPSI score accuracy.

## Conclusion

A single intralesional injection of BoNT-A offers results comparable to multiple sessions of TA injections, demonstrating prolonged efficacy, particularly in lesions involving nail bed pathology. Consequently, BoNT-A injection emergers as a promising and novel treatment option for nail psoriasis.

## Conflicts of interest

None disclosed.
